# Making future udders: Mammary development and perinatal programming of dairy cattle

**DOI:** 10.3168/jdsc.2025-0828

**Published:** 2025-08-28

**Authors:** Jimena Laporta, Maverick C. Guenther

**Affiliations:** Department of Animal and Dairy Sciences, University of Wisconsin–Madison, Madison, WI 53706

## Abstract

The perinatal period, described herein as the time spanning the final 2 mo of gestation through 2 mo postnatal, is a critical window of developmental plasticity for many organs in placental mammals, including the mammary gland (MG). In dairy cattle, early-life MG development involves foundational morphogenic events that are highly sensitive to environmental and nutritional factors. Emerging evidence challenges the long-standing belief that substantial MG development begins after weaning and lasts until puberty, showing instead that preweaning mammary parenchymal (mPAR) growth is allometric and its degree of development can influence future lactational capacity. Overall BW typically doubles from birth to 60 d, whereas both whole udder weight and the mammary fat pad increase by ∼2.2 to 3 times over the same period. In contrast, the mPAR exhibits pronounced allometric growth, expanding 15 to 35 times during this time. Early-life management strategies, such as heat abatement to maintain thermal homeostasis and enhanced nutrition through higher milk intake supporting greater average daily gain, further promote mPAR development and can positively influence future lactation performance of dairy cows. These findings underscore the importance of integrating developmental biology into heifer-rearing strategies and emphasize the need for precise environmental and nutritional management during this critical window to support lifelong mammary function and optimize herd performance.

## Highlights

• The perinatal period is a critical window for mammary parenchymal development.
• Early-life heat abatement and enhanced milk nutrition promote parenchyma growth.
• Environmental and nutritional factors can program future lactational performance.

## Graphical Abstract

**Figure:**
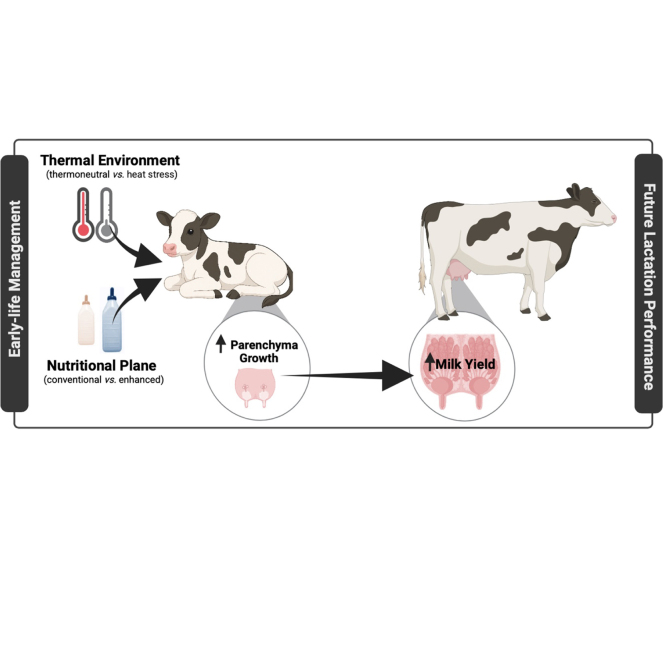
**Summary:** The last 2 months before birth and the first 2 months after birth represent a crucial window for organ development, including the mammary gland, in dairy cows. Traditionally, it was thought that allometric mammary growth began after weaning or around puberty. However, recent research indicates that significant early development of the mammary parenchyma occurs during the perinatal period and can influence a cow's future milk production. This early growth is highly sensitive to environmental and nutritional factors. Heat stress abatement and higher nutritional planes in early life increase mammary parenchyma growth and improve future productivity. Proper management during the perinatal period is essential to optimize lifetime milk yield.

Perinatal programming—the concept that environmental conditions during critical windows of early development can shape long-term physiological outcomes—has become a foundational principle in developmental biology. This concept was first recognized in humans through the epidemiological work of David Barker linking poor maternal nutrition during the Dutch Hunger Winter to adult-onset of cardiovascular disease (Barker, 1990). Since then, this idea has expanded into the animal science field. Strong evidence now supports that the late gestation and early postnatal period—collectively referred to herein as the perinatal window—represents a critical time of developmental plasticity. Developmental plasticity during this time allows the fetus and neonate to adjust their physiology, metabolism, and organ development, anticipating the postnatal environments or adjusting to the current environment conditions, respectively. This adaptive capacity helps them optimize the allocation of resources to support survival, growth, and the functional maturation of key organ systems. Developmental plasticity adaptations are driven by intricate interactions between environmental cues, such as temperature, nutritional status, and perceived stressors, which influence gene expression patterns that regulate the growth, development, and functional differentiation of organs (Bell and Greenwood, 2016).

Adverse in utero conditions, including maternal nutrient restriction and hyperthermia, can alter normal fetal growth trajectories, often resulting in asymmetric development that prioritizes critical organs like the brain over peripheral tissues in various livestock species (Reynolds et al., 2023; Laporta and Skibiel, 2024). Although developmental adaptations to insults may enhance short-term survival, they often come at a cost, potentially leading to decreased immune competence, suboptimal growth, and reduced productivity later in life. Following birth, the early postnatal period, particularly the first 2 mo of life, remains a highly plastic developmental window during which several organ systems are still maturing and therefore susceptible to environmental and nutritional influences. In livestock species, immune, metabolic, endocrine, and exocrine organs continue rapid structural and functional development during this early life phase. Adverse external factors—such as undernutrition, thermal stress, and pathogen exposure, among others—have been shown to program lasting physiological and structural outcomes in dairy cattle and other species. For example, lack of colostrum ingestion is linked to poor maturation and development of the gastrointestinal tract (Pyo et al., 2020). Additionally, postnatal heat stress negatively affects development by impairing liver homeostasis, altering mammary gland (**MG**) growth, reducing milk yield, and affecting survivability to the first lactation (Guenther et al., 2025; Laporta et al., 2025).

In contrast, specific stimuli can help mitigate early-life stressors and support healthier developmental trajectories of key tissues and organs. Growing evidence in livestock species demonstrates that the early-life environment can epigenetically and physiologically program animals leading to positive long-term phenotypic effects (Laporta et al., 2022; Vautier and Cadaret, 2022). When these conditions are favorable, such as through adequate heat abatement interventions to maintain thermal homeostasis, developmental adaptations can promote optimal organ maturation, enhance metabolic efficiency, and induce beneficial epigenetic modifications (Laporta et al., 2025) that may be stable until adulthood (Skibiel et al., 2018). In line with this, an adequate nutrient supply (higher planes of nutrition) during critical development windows leads to epigenetic alterations that change gene expression (Vailati-Riboni et al., 2018), and these changes may persist and influence long-term physiology (Loor, 2022). These physiological and molecular changes ultimately prepare the animal for improved health, greater resilience to stress, and enhanced productivity, underscoring the lasting influence of early-life conditions on long-term performance.

A key organ of immense relevance to the dairy industry and the primary focus of this review is the MG. The bovine MG undergoes a sustained period of development and uniquely experiences phases of growth and regression across the animal's lifetime (Hurley and Loor, 2011; Akers, 2017). Although the majority of its functional growth takes place postnatally, MG development begins much earlier (in utero) and continues through the early preweaning phase before reaching the pubertal, gestational, and lactation phases of development characterized by substantial systemic endocrine changes (Figure 1). Mammary growth and development across these different stages can be characterized by macrostructural changes, such as udder enlargement driven by mammary fat pad (**mFP**) expansion, as well as microstructural changes, such as lobulo-alveolar growth and differentiation within the mammary parenchyma (**mPAR**). Notably, macrostructural growth without proper microstructural maturation would result in a gland that appears developed but lacks the cellular foundation to support lactation. Understanding early-life mammary growth and its susceptibility to environmental factors during the perinatal period offers an opportunity to refine management strategies that optimize lifetime milk yield.

**Figure 1:**
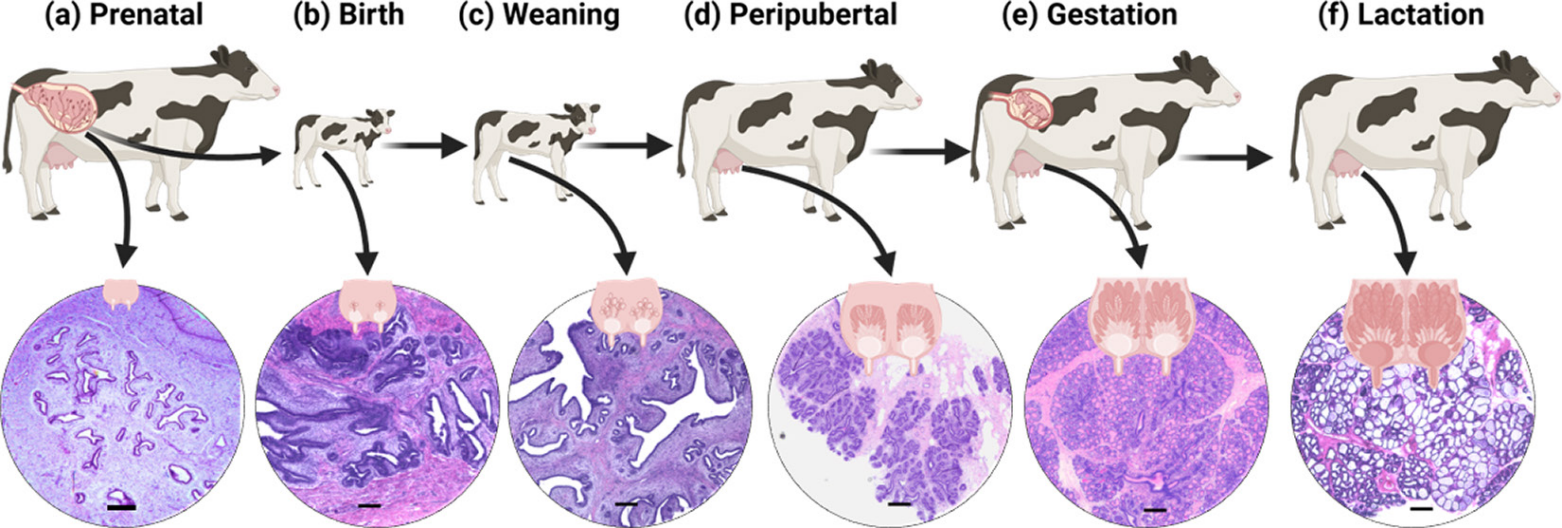
Major longitudinal macro- and microstructural development of the bovine mammary gland from the prenatal period to first lactation. Mammary development begins prenatally (in utero; a) and progresses postnatally through key stages: (b, c) preweaning (birth to 2 mo), (d) postweaning to puberty (∼2–13 mo), (e) gestation (∼13–24 mo), and (f) first lactation (∼24–34 mo). Ductal growth is initiated during the perinatal period (−2 to +2 mo relative to birth), although functional alveoli have not yet formed. This critical developmental window establishes the mammary fat pad, parenchymal mass, and the initial epithelial-ductal architecture, laying the structural foundation for future mammary growth, differentiation, and lactation performance. Representative hematoxylin and eosin-stained sections of mammary tissue shown at (a) the prenatal stage (150 d of gestation; scale bar = 200 μm; Hara et al., 2018; reproduced from Hara et al., J. Vet. Med. Sci. [2018]. ©Japanese Society of Veterinary Science. Licensed under CC BY-NC-ND 4.0.); (b, c) birth and weaning (4 h of life and 63 d of age; scale bar = 500 μm; Dado-Senn et al., 2022); (d) peripubertal stage (12 mo of age; scale bar = 500 μm; Davidson et al., 2025); (e) gestation (6 mo pregnant; scale bar = 500 μm, J. Laporta, unpublished); and (f) lactation (42 DIM; scale bar = 500 μm; Skibiel et al., 2018).

Each developmental stage of the MG plays a critical role in shaping a heifer's future milk production potential. Specifically, prenatal mammary development begins early in gestation with the formation of the mammary line and bud at ∼35 and ∼45 d of gestation, respectively (Akers, 2002). These structures continue development throughout fetal life with elongation and branching of primary and secondary ducts (Hara et al., 2018). At birth, the MG consists of an immature mPAR with rudimentary ductal epithelial systems which are encased in stromal tissue. This mPAR structure will begin to invade the surrounding mFP. These early morphogenic events establish the architecture of the gland for future development (Knight and Sorensen, 2001). Historically, the preweaning period—from birth to ∼8 wk of age—was considered a relatively inactive phase for mammary development, with growth believed to occur isometrically, at the same rate as overall body growth. This view was based on studies that assessed the whole gland macrostructure and DNA or RNA content as proxies for growth. However, recent studies using immunohistochemistry and gene expression analysis on specific tissue types have revealed substantial microstructural and molecular changes during this period.

Although studies during this early developmental stage are still limited, key findings have demonstrated that mPAR volume increases markedly, with up to a 60-fold increase from 30 to 90 d of age (Akers et al., 2005). Based on ultrasound measurements, a 6-fold increase in the cross-sectional area of mPAR from wk 1 to wk 8 of life was reported (Esselburn et al., 2015). In a recent study, we euthanized and dissected udders of Holstein heifers at birth, revealing a 25-fold increase in mPAR mass from birth to 63 d of age, compared with 2.1- and 2.3-fold increases in the mFP and whole udder, respectively (adapted from Dado-Senn et al., 2022; Figure 2). This mPAR growth far exceeds the 2.2-fold increase in BW, highlighting the allometric nature of development during this time. Moreover, epithelial and stromal cells in the mPAR exhibited substantially higher proliferation rates at birth and weaning compared with early lactation mammary tissue (Figure 3). The rapid growth of the MG at this time is influenced by a complex interplay of systemic and locally produced hormones including estrogen, growth hormone, and IGF (Akers 2017). These findings underscore the biological importance of the preweaning period in establishing mammary developmental potential, highlighting that any perturbations during this critical period may disrupt the foundational growth and proliferation processes. After weaning through puberty, the whole gland undergoes continued allometric growth. Estrogen and progesterone promote ductal elongation, branching, and early alveolar development. The most profound transformation occurs during gestation, with synergistic effects of estrogen, progesterone, and prolactin stimulating lobulo-alveolar proliferation. These changes culminate in lactogenesis at parturition, driven by hormonal shifts (Tucker, 2000). The degree of mammary development at calving determines peak yield and lactation persistency (Capuco and Choudhary, 2020), making these times of early development a key determinant of lifetime productivity.

**Figure 2:**
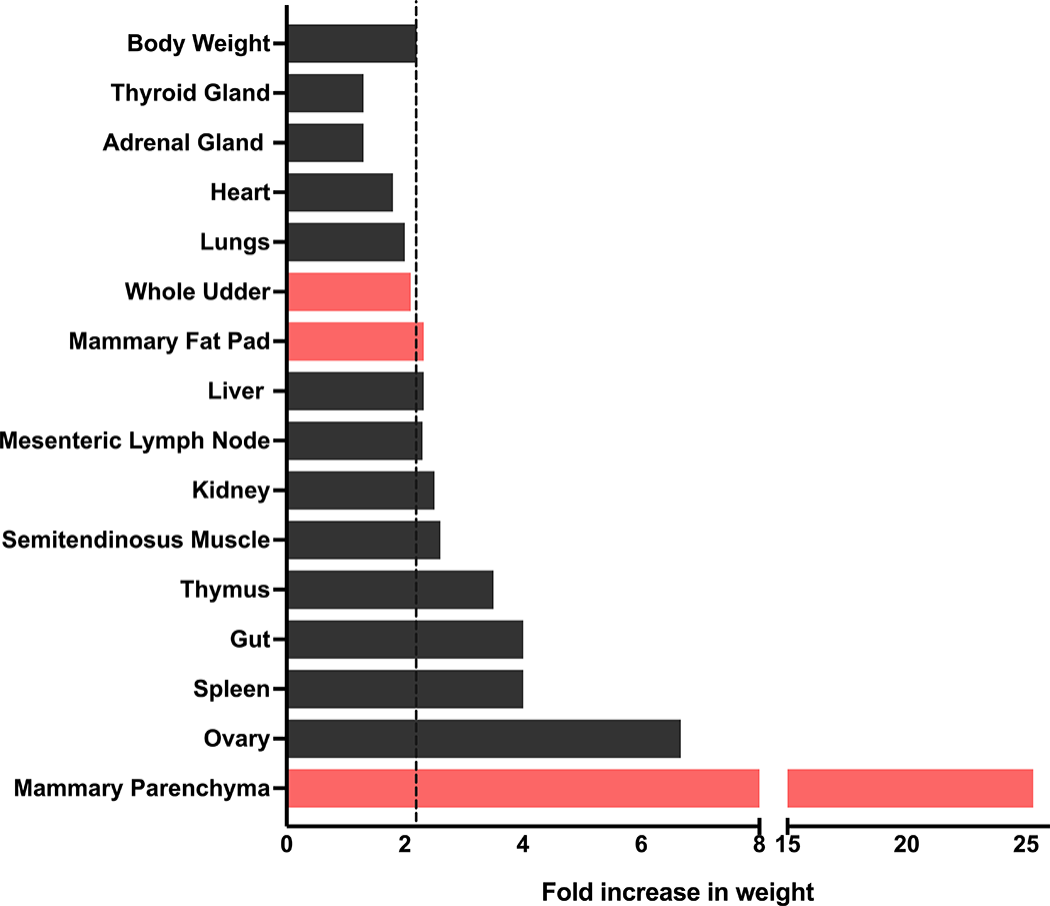
Body and organ growth from birth to weaning in Holstein heifers. The dotted vertical line represents the change in live BW during the preweaning period (0–63 d of age, 2.2-fold increase). Bars illustrate the change in absolute organ weight (Dado-Senn et al., 2021) over the preweaning period, with pink bars denoting mammary gland components (whole udder, mammary fat pad [mFP], and mammary parenchyma [mPAR]; Dado-Senn et al., 2022). The mPAR demonstrates pronounced allometric growth (25-fold increase), whereas the whole udder and mFP exhibit isometric growth (2.1–2.3-fold increase), proportional to BW. These findings emphasize the disproportionate expansion of the epithelial-rich mPAR relative to other mammary and extra-mammary tissues during early postnatal development.

**Figure 3:**
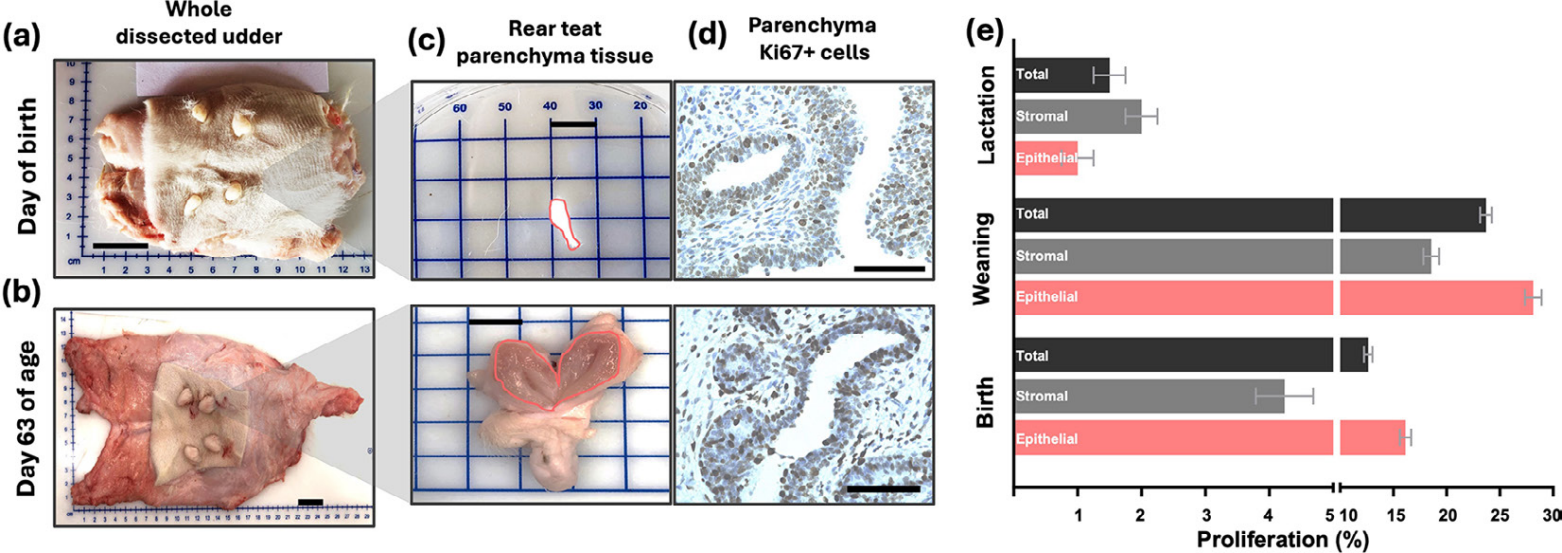
Mammary gland development from birth to weaning and proliferation at birth, weaning, and lactation. Gross morphology of the whole dissected udder was assessed (a) 4 h after birth and (b) at 63 d of age (scale bars = 2.4 cm). (c) Representative images of mammary parenchyma (mPAR) dissected from the region proximal to the rear teat (scale bars = 1 cm). At 63 d, the mPAR was bisected and the teat was left for visual reference. (d) Corresponding immunohistochemistry images showing Ki67^+^ (brown) cells, used to assess epithelial cell proliferation (scale bars = 100 μm). (e) Quantification of epithelial (pink bars), stromal (gray bars), and total (black bars) proliferative index (% Ki67^+^ cells) in mPAR tissue at birth, weaning (0 and 63 d of age; Dado-Senn et al., 2022), and early lactation (21 DIM; Skibiel et al., 2018). The average total proliferation indices were 13%, 24%, and 1.5%, respectively. Data are presented as mean ± SEM.

Environmental and nutritional conditions experienced by dairy heifers during the perinatal period can exert long-term effects on future production capacity by programming gene expression patterns that shape the developmental trajectory of the MG. Early-life hyperthermia during the perinatal period can impair the growth and maturation of the MG due to the disruption of hormonal signaling, cell proliferation, vascular development, and epigenetic programming that normally occur in a thermoneutral environment. Specifically, prenatal heat stress during the final 2 mo of gestation leads to lighter calves with reduced mFP and mPAR at birth, independent of birth weight (Dado-Senn et al., 2021, 2022). Ductal structures in prenatally heat-stressed calves are underdeveloped at birth and remain smaller with substantially less branching at weaning. Although mPAR mass (in terms of size and weight) recovers by weaning, the mFP pad does not, potentially limiting subsequent mPAR invasion and impairing further mammary development (Dado-Senn et al., 2022). Cell proliferation within ductal epithelium is essential for mammary tissue expansion, as it drives the growth and elongation of the ductal network. Notably, heat stress in utero reduced cell proliferation by ∼50%, indicating persistent morphogenic impairments that are evident at birth and weaning (Dado-Senn et al., 2022). However these reductions also extend into puberty and the first lactation, with reduced lobulo-alveolar development and a 25% to 30% lower mammary epithelial cell proliferation rate in glands of prenatally heat-stressed heifers (Skibiel et al., 2018; Davidson et al., 2025). Ultimately, heat stress exposure during the late stages of fetal life derails mammary development, leading to lasting microstructural changes in the MG that result in lower synthetic capacity at maturity (Monteiro et al., 2016; Laporta et al., 2020). Conversely, providing heat abatement to pregnant cows during hot summer months allows late stages of gestation to occur under thermoneutral conditions, which has been shown to reverse the detrimental effects triggered by in utero hyperthermia on postnatal whole body and MG development (Davidson et al., 2025). Specifically, heifers born to cool dams have larger udders with a 2-fold increase in the mPAR mass at birth, with restored cell proliferation when assessed longitudinally (Dado-Senn et al., 2022; Davidson et al., 2025). By preserving mPAR growth during late gestation, prenatal heat abatement supports long-term mammary function and enhances lifetime milk production (Laporta et al., 2020).

Thermoregulation of neonatal mammals, including calves, is immature at birth, rendering them particularly susceptible to environmental heat stress, which may perturb hormonal and metabolic pathways that affect the MG. Although direct studies specifically examining postnatal heat stress during the first months of life and its long-term impact on MG development in livestock species are limited, evidence from our group supports that preweaning heat stress in dairy calves alters MG development (Guenther et al., 2025). Ongoing studies are investigating the long-term effects of preweaning heat stress to understand if these changes lead to decreased milk production in adulthood. Briefly, preweaning heat stress exposure in Holstein dairy heifers results in the development of disproportionately larger udders harvested at 63 d of age, driven by an expansion of the mFP rather than mPAR tissue. Despite similar overall mPAR weight and size compared with thermoneutral counterparts, calves exposed to heat stress exhibit significantly reduced cellular proliferation within the mPAR, an essential process for ductal and lobulo-alveolar development, potentially compromising later stages of mammary development and their future milk-producing capacity (Guenther et al., 2025). This evidence underscores the critical importance of managing stressors in early life, ultimately promoting mammary health and optimizing lifelong lactational performance.

Historically, rearing strategies have prioritized rapid growth at minimal cost to minimize the nonproductive lifespan of heifers, often at the expense of optimal organ development. However, this paradigm is beginning to shift as evidence is accumulating in favor of more biologically appropriate, nutrition-focused management approaches that support long-term productivity. Enhanced nutrition during the preweaning period—particularly increased milk or milk replacer intake—supports greater ADG and stimulates mammary development. Comparisons across studies providing different planes of nutrition in early life can be challenging, as some studies alter the level of milk allotment (Daniels et al., 2009; Soberon and Van Amburgh, 2017; Niwińska et al., 2022), milk replacer composition (Meyer et al., 2006), or both allotment and composition (Brown et al., 2005; Daniels et al., 2009; Geiger et al., 2016). However, across the previously noted studies, which harvested and dissected mPAR tissue around weaning, enhanced planes of nutrition result in a 2.3- to 6-fold increase in mPAR mass with a 1.1- to 1.4-fold increase in BW. Enhanced nutritional input during the preweaning phase has also been associated with increased mammary epithelial cell proliferation and increased mammogenic hormone receptor abundance within the mPAR (Geiger et al., 2017). Additionally, an enhanced plane of nutrition during the preweaning period increases circulating IGF-I (Rosadiuk et al., 2021), which has been implicated as a key hormone for MG development (Akers et al., 2000). This demonstrates that despite the differences in feeding management across studies, mPAR tissue is highly responsive to improved nutrition during this critical developmental window.

Although a clear knowledge gap remains regarding the early postweaning period and its influence on mammary development and future lactation performance, meta-analyses have linked preweaning enhanced ADG to improvement in first-lactation milk, protein, and fat yield (Soberon and Van Amburgh, 2013; Jiang et al., 2025). More specifically, each additional kilogram of preweaning ADG was associated with ∼1,550 kg more milk in the first lactation (Soberon and Van Amburgh, 2013). In a separate meta-analysis, studies supported the notion that higher preweaning ADG (0.6–1.0 kg/d) significantly increased 305-d milk, fat, and protein yield, with the strongest positive associations with first-lactation performance observed as ADG approached the upper end of this range (Jiang et al., 2025). Interestingly, starter DMI showed no significant associations with any first-lactation performance metrics (Jiang et al., 2025). Together, these findings underscore the critical importance of early-life nutrition in determining not only overall growth, but also the developmental trajectory of key tissues like the MG leading to improved lactation.

In summary, this review provides evidence that mPAR development is allometric, highly proliferative, and developmentally plastic during the perinatal period, and that this initial growth phase may be critical in shaping future lactational capacity. Despite growing evidence in support of early-life management influencing MG development, few controlled long-term studies link these changes to future lactation, limiting our ability to assess lasting impacts. Our rapidly evolving understanding underscores the need for science-driven, proactive heifer-rearing strategies that recognize the long-term implications of early-life exposures. Optimizing environmental and nutritional conditions during these sensitive windows of development will likely improve productivity, resilience, and welfare in future dairy herds.
